# Hospital Meetings, &c.

**Published:** 1898-12-10

**Authors:** 


### The Institutional Workshop.

### HOSPITAL MEETINGS, &C.

### ADDENBROOKE'S HOSPITAL.

Adjourned Meeting.

The adjourned meeting of the governors of Addenbrooke's Hospital, Cambridge, for the purpose of further considering the recommendations of Sir Henry Burdett on the management of the hospital was held on Monday, the 5th inst. Mr. Hamblin Smith was voted to the chair.

The Secretary (Mr. Bonnett) reported the result of the poll taken upon the first of the series of recommendations of the Special Committee, which we gave in last week's issue of The Hospital.

Mr. Clay said that before proceeding with the business he wished to be allowed to make a few remarks. Up to the last meeting he had not read the Act of Parliament, but since then he had read it carefully through, and he had come to the conclusion that the Special Committee had not given attention to the fundamental principles of it. (Hear, hear.) It was laid down in the Act of Parliament that a general court of the governors should be held at the hospital four times at least in every year, or oftener if occasion should require, upon notice thereof being given. At such courts all bye-laws could be revoked. It was manifestly intended that the Committee of Management must be subordinate to the General Court, which alone had authority to enter into contracts. No committee, however appointed, could over-ride the General Court, and it was manifest that all their actions must be controlled by such court, meeting quarterly or oftener, as might be required. No resolution could take away the rights of the governors meeting at these courts, which had full power to overhaul the business of the Weekly Board. The only way in which any alteration in the procedure could be effected was by the abrogation of the existing Act of Parliament and the enactment of another in its place. (Hear, hear.)

The Chairman said he presumed it was intended that the recommendations of the special committee should be taken in order. He proposed to adopt that course. He would, however, make this remark with regard to any particular resolution. It should be regarded in two aspects: First, could the thing recommended be done? and secondly, was it desirable that it should be done? With regard to the first point, the remarks of Mr. Clay were pertinent, and led up to what he (the chairman) had to suggest, viz., that they should at some juncture of the proceedings pass a resolution empowering the committee to obtain legal advice—(hear, hear)—in order that they might be quite sure that any bye-law that it was felt necessary to make did not contravene either the letter or the spirit of the Act of Parliament. In recommendation four the important point was as to whether a selection of a committee could be made, but when they had settled that they had to consider whether it was desirable that the number should be as large as recommended. The same thing occurred with regard to the financial portions of the scheme. He had to make one more remark. It was very desirable, in his opinion, that those who spoke should as far as possible confine themselves to the particular recommendation under discussion. (Hear, hear.) They would render a great service to the committee if they would in the first place point out where these recommendations contravened the Act, and, secondly, give their reasons why the recommendations should or should not be adopted.

Dr. MacAlister said the chairman had anticipated very much what he was going to say. When they passed a resolution that a certain thing was desirable, it was not operative until bye-laws had been framed and passed and confirmed by the General Court. If the General Court chose to pass a bye-law which was illegal, it would, *ipso facto*, be null and void. But the fact that the resolution had been passed was not touched by what Mr. Clay had said. When they got to No. 10 of the recommendations he should propose to add to it the following words: "That the Special Committee be instructed to take legal advice as to whether or not it is within the power of the governors to pass it."

Dr. Waraker rose to a point of order. Mr. Clay had already anticipated what he was about to say.

Mr. Parker: What is before the meeting?

Dr. Waraker: I rise to a point of order. Continuing, Dr. Waraker said Dr. MacAlister had told them that the vote was only provisional, but the resolution which had been passed was that a committee of management, together with the chairman, should be appointed annually, whose duties should include those at present assigned to the Weekly Board, the Select Governors, and the Finance Committee. He took it there were certain functions prescribed in that recommendation; it was not merely a recommendation of expediency, but an absolute proposition, which was carried last Monday. Dr. MacAlister had said that these things could not come into force until new bye-laws had been passed. That was not the case. It was not the bye-laws which fixed the position of the governors; their position was fixed by statute. (Hear, hear.) Dr. Waraker was proceeding to read the powers conferred by the Act,

When Mr. Parker rose to a point of order.

The Chairman (to Dr. Waraker): What resolution are you going to propose?

Dr. Waraker replied that, he was going to propose that, in view of the doubts entertained, the secretary be instructed to obtain the opinion of counsel on the question.

The Chairman remarked that none of these resolutions could take effect until a court was held to consider the bye-laws framed to carry them out, and it struck him that would be the occasion for the discussion on the intention of the Act. In other words they would then have a larger audience and a more competent chairman. ("No, no!") At present he asked them to regard the meeting as one for the dispatch of business and the passing of these resolutions.

Dr. Waraker said that with all due submission he would suggest that was precisely his purpose, because if it were held that the governors had no right to pass such a recommendation as had been passed, the whole thing would fall to the ground. Whatever might be the result of submitting the case to counsel, it would at all events require only one discussion. The discussion that day would be idle because it might be held——

The Chairman: You are referring to the first resolution, which has already been passed. I do not think this court is competent to alter resolutions.

Dr. Waraker replied that they could not consider that it was passed. What he said was that it was *ultra vires* and illegal, because it interfered with the rights of the governors, which were settled by statute.

The Chairman said these were remarks which should be made later. (Applause.)

Dr. Latham said if the governors had done anything *ultra vires*, and passed a resolution which could not be enforced, it would be better to adjourn the meeting and take counsel's opinion upon the point before they proceeded any further, because, if this resolution was *ultra vires*, other resolutions dependent upon it were entirely out of place. He thought it would be better to hear the opinions of the various lawyers present before they proceeded any further.

Sir Henry Burdett rose to a point of order. Some of them, he said, were there at great inconvenience to discuss this series of resolutions, of which due notice had been given. That was their business, and the meeting had been specially called for that purpose. As to the legal aspect, that had been dealt with in a proper way by the chairman. He was responsible for the principles underlying these recommendations, and he had not ventured to make the recommendations which were found in his report without taking the opinion of one who had had much to do with this kind of work. He had consulted Mr. Bristowe, and all these recommendations, according to his judgment and opinion, were within the constitution. He thought, therefore, it would be altogether out of order not to proceed with the ordinary business. When they got through the recommendations the legal point would come up. But, as the chairman had rightly said, when the entire scheme, of which the governors might approve, was determined, then they would have the whole case in a concrete form to submit to counsel. If they did not come to a decision upon the other recommendations the case could not be complete.

Dr. Waraker (to Sir Henry Burdett): Have you Mr. Bristowe's opinion with you?

Sir Henry Burdett: No, I have not got it with me.

Dr. Waraker: Have you the case?

Sir Henry Burdett: The case is the report.

Dr. Waraker: Have you the case on which Mr. Bristowe based his opinion?

Sir Henry Burdett: No, I haven't it here.

Dr. Waraker: Then we only have Sir Henry Burdett's statement that some sort of case was submitted to Mr. Bristowe. (Cries of "Order, order!" and dissent.)

### Counsel's Opinion.

The following is a copy of Mr. L. S. Bristowe's opinion referred to by Sir Henry Burdett:—

"Under Addenbrooke's Hospital Act the Governors have power (page 14) 'To appoint such weekly or other meetings of the said Governors or any five or more of them with such powers and authorities as they shall think necessary.' I think this is a general power to appoint committees of not less than five Governors for the transaction of any business not expressly required to be done at a General Court.

"Independently of the provision on p. 16 of the Act authorising casual vacancies to be filled by a committee (which is limited to vacancies caused by death, suspension, or removal) I think the Governors can, under the general power above mentioned, constitute an Election Committee with the duty of investigating the qualifications of candidates for any vacancy and selecting one for recommendation to the next General Court. And even if the vacancy were caused otherwise than by death, suspension, or removal and the well-being of the hospital required it I do not think that there would be any objection to giving the Election or any other Committee power to nominate a person (who might be the selected candidate) to discharge the duties of the office pending the next General Court.

"The actual appointment is expressly required to be made at a General Court (p. 16) and could not in my opinion be delegated to a committee. "(Signed) "L. S. Bristowe.

"1, New Square, Lincoln's Inn, 18th June, 1898."

Mr. Clay: Dr. Waraker is quite in order.

Dr. Waraker (to the Chairman): I must ask you to protect me from these extremely unseemly interruptions. (Hear, hear.) I say that this first recommendation is one that carries all the rest with it, and it would be a saving of time to everybody if the legal propriety of the recommendation were submitted to counsel. (Hear, hear.)

The Chairman: I shall rule that this is a matter which is not under the consideration of this court. We will proceed with the second resolution.

Dr. Waraker: Do you refuse to hear me?

The Chairman: I think I have heard you.

Dr. Waraker: No. I was going to demonstrate that this is wholly illegal. I repeat it, and I protest against it. (Hear, hear.)

The Rev. G. B. Finch proposed: "That this court stand adjourned until the opinion of counsel on the whole of the recommendations proposed by the committee has been obtained." That was a proposal which was quite in order. (Applause.)

The Chairman: You are not following my ruling at all. I propose we should go on with number two.

The Rev. G. B. Finch: I propose that we adjourn. Proceeding, Mr. Finch said he would show why he took such an extreme course. He was perfectly convinced that it was absolutely wrong to attempt to vest in any part of the governors the jurisdiction of the whole. His next point was that the general board should appoint such weekly or other meetings of the said governors, or any number of them, with such powers as they thought necessary for the easier execution of the Act. The operative part of that was whether there should be weekly meetings, fortnightly, or any other term, but it said there should be such and such meetings, and any governor might attend. There was nothing to prohibit that. There was the legal right of every governor to take part in the administration of the hospital, and there was nothing whatever in direct terms to disfranchise any governor. Therefore, what was the good of these administrative changes if they were illegal. The chairman had suggested that the time for considering whether this resolution that had been passed was legal or illegal was when they came to pass the bye-laws. It would then be too late. With such misgivings which he seriously and honestly entertained, he had no hesitation in asking that meeting to adjourn. With regard to Sir Henry Burdett, they could not expect the gentlemen present should always acquiesce in his statement of the case and his selection of counsel; but they would all like to have a voice in drawing up the statement and in the framing of the case. With no disrespect to Sir Henry Burdett, they would like to have the matter in their own hands, and he was sure Sir Henry could not object to that.

The Chairman: I suggest to you that we should proceed to recommendation 2, but it has been proposed that we should do no such thing.

Dr. Latham said he had a great deal of pleasure in seconding the proposition of Mr. Finch.

The motion for adjournment was carried, 19 voting for and 17 against it.

The Chairman said perhaps before the meeting adjourned they might make some suggestion of what they proposed to do.

Dr. MacAlister remarked that the governors had now involved themselves. He had not heard the resolution read out before they voted, and he thought they had voted under a wrong impression.

The Rev. G. B. Finch: It was read out.

Dr. Latham remarked that it was out of order to discuss that now.

The Secretary informed Dr. MacAlister that he had read the resolution before it was voted upon.

Dr. Latham proposed that a committee of one gentleman from each side be appointed to draw up and submit the case to counsel. He would like to propose Mr. Finch, if that gentleman was willing.

The Chairman: Don't you think it ought to be presented to the committee?

Dr. Latham: Not at all. I think it ought to be two independent gentlemen. I leave Dr. MacAlister to nominate one of the other side.

The Rev. G. B. Finch remarked that he did not think they could do that. He thought the proper course would be for the secretary to prepare the case and bring it before the court to see that it was a proper representation.

Sir H. Burdett said they had unanimously elected a special committee to take into consideration certain matters. Their report was now before the governors, and to appoint another committee was to supersede the sub committee and pass a vote of censure upon them, though he did not think that was the intention of the governors.

The following resolution, "That the secretary take the opinion of counsel on the whole of the recommendations, with the assistance of the Rev. G. B. Finch and Mr. Parker, and report to a meeting to be fixed on December 26th," was carried.

On the motion of Professor Bradbury, seconded by Mr. Peck, it was decided that the statement of the case and counsel's opinion should be printed and circulated among the governors.

The meeting then terminated.

### OPENING OF ELTHAM COTTAGE HOSPITAL.

A large company of ladies and gentlemen assembled on Saturday afternoon at the new Eltham (Kent) Cottage Hospital, to witness the opening by Sir Henry Burdett, K.C.B. The new hospital, which has been erected on an admirable site, having a wide open space all round it, and yet within a very short distance of the High Street, is a pretty building. It is built on the pavilion system, and the whole of the administrative block is entirely apart from the wards. There are two principal wards, which will accommodate ten patients, and there are two private wards, bath-room, &c., and spacious corridors. The architect is Mr. A. B. Hutchings, 14, Victoria Street, Westminster.

Among those present at the opening ceremony were: Sir Henry Burdett, Colonel H. M. Gordon, J.P. (chairman of the Building Committee), Mr. T. W. Mills (hon. secretary), Mr. W. H. Richardson, the Rev. T. N. Rowsell, the Rev. E. Martin, the Rev. E. J. Penford, the Rev. G. B. P. Viner, Mr. David French, Mr. James Spicer, &c.

Colonel Gordon presided, and said as chairman of the Building Committee it was with much satisfaction that he was able to congratulate the parishioners upon the completion of that work. They must all be aware of the circumstances which led to the erection of the hospital—the unsuitableness of the old dwelling-house that formerly served as a hospital, and the opportunity that the Queen's Diamond Jubilee gave for raising funds to erect this new and worthier building. Colonel Gordon then referred to the very generous offer of £500 from Mr. F. G. Saunders, which formed the nucleus of a building fund, after which the scheme was enthusiastically taken up, with the result they saw before them. They were not opening entirely free from debt, as a sum of £500 to £600 is still required to free them from all debt. They welcomed Sir Henry Burdett there that day to open the hospital. (Applause.) Sir Henry Burdett was the greatest authority in Great Britain upon cottage hospitals, and his book upon "Cottage Hospitals," the first edition of which was published more than twenty years ago, had given the committee many valuable helps in building, as he was sure it would continue to do in the future management of the hospital.

Sir Henry Burdett said it gave him great pleasure to open that, the latest cottage hospital which had been erected for the comfort and cure, not only of the sick poor, but of all classes of the community. He would wish to earnestly impress that fact upon them, because it was too often forgotten when people were asked to give, that by so doing they helped to make a provision for their own time of sickness or accident or the illness of those dependent upon or dear to them. It was remarkable that forty years ago there were no cottage hospitals in this country. The mere mention of that fact would bring vividly to their minds how rapidly we had moved in these latter times, and how great had been the changes, which were largely due to the enormous increase and growth of our population. When they realized that every year the increase in the population of London amounted to a figure which in itself represented a great city, viz., some 80,000, and that the population was increasing throughout the country, they must see the necessity of increased hospital accommodation. Referring to the origin of cottage hospitals, Sir Henry said forty years ago a country surgeon, Mr. Napper, felt that he could not do justice to his wealthier patients and provide properly for the alleviation of suffering, unless he had some hospital accommodation in his own village. The proposal was objected to by those who were responsible for the conduct of the county hospital, because, they argued, if Mr. Napper succeeded in establishing a cottage hospital, and the system grew, the county hospitals must suffer, not only in patients, but also in cash. Indeed, into whatever district this movement of establishing cottage hospitals had been carried, there was at first great opposition to it, and even up to quite recent years the same arguments had had to be combated and the same difficulty had been experienced. But they had survived such opposition, and cottage hospitals to-day were universally admitted to be the right thing, and why? It was because they met a public necessity. Looking at the number of cottage hospitals in the country to-day, and looking at the beneficent work accomplished by these hospitals, one must be struck with thankfulness that wherever they had been established they had been the means of alleviating suffering and saving life amongst rich and poor alike.

### The Practical Value of Cottage Hospitals.

Let them think for a moment of the usefulness of a cottage hospital. Wherever there was a pack of hounds there was the risk of accident. In such cases the cottage hospital was at hand, and the sufferer received the prompt aid of the local surgeon, who gave skilled treatment, because he had been able to "keep his hand in," so to speak, at the cottage hospital. He (the speaker) had personally cause to be thankful to cottage hospitals. It so happened that one of his boys was at Marlborough College when the fact of four Unionist candidates being returned for the county of Wiltshire, for the first time, was causing a good deal of excitement. And the boys, catching the enthusiasm, unhorsed the carriage of one of the newly-elected members and drew it round the "quad." Unhappily, one of the ropes gave way, and about twenty little fellows were thrown down, and his own boy's head became a skid for one of the wheels. The injuries were so fearful that the boy was thought to be killed, and had it not been for the fact that there was the Savernake Cottage Hospital near by, with the trained skill and untiring attention of Dr. Morris and his son, with Dr. Penny, he might possibly have died, or certainly have been disfigured for life. Happily these gentlemen were at hand, and the boy made a complete recovery. That was one result of the existence of cottage hospitals, and the skill of the surgeons who were always in ready attendance at these institutions. He asked those who had taken no interest in cottage hospitals in the past to do so from to-day. He must be a strange being who was not moved to sympathy and liberality when he considered what a hospital meant to everybody in a community, and especially to the humbler folk. Having regard to the position now occupied by the cottage hospital movement, it was small wonder that the idea had extended to the United States of America, cottage hospitals being now established throughout Maryland and Massachusetts. In England we had a great many medical men, and one or more in each village acted as the honorary medical officer of the cottage hospital. The valuable and unostentatious honorary work done by these medical men was universally admitted, and it had long struck him as an anomalous position that whereas it was the custom, and had been the custom for centuries, to select for honour certain leaders of the medical profession who were attached to large hospitals, there had never been any distinction offered to a cottage hospital surgeon. He did hope—and he spoke with some knowledge of the state of feeling on this subject—that we might be near the time when the beneficent work of cottage hospital surgeons throughout the country would be recognised by an honour to one of its representatives, because of the important bearing it would have upon the whole position and work of what constituted a very large proportion of the medical profession. (Applause.) He did think it would be a very popular and very wise act if the present Prime Minister were to make a selection of one or two gentlemen who had devoted their lives as medical officers to cottage hospitals for some distinction which should mark his sense and Her Majesty's sense of the importance of these gentlemen's services to the community. (Applause.)

### The Eltham Cottage Hospital.

Turning to the hospital he attended to open to-day, Sir Henry Burdett said he remembered Eltham twenty-five years ago, when he used to come there because of its pleasant lanes and fields, and he had been interested to-day to notice certain changes that had taken place, not the least important of which was the erection of this hospital on so admirable a site. (Applause.) Many years ago, when a student at Guy's Hospital, he remembered cases of accident being brought up from this district, and he had known deaths to have occurred in consequence of the long distance that had to be traversed, over rough roads, in unsuitable conveyances, before they could receive hospital treatment. They in Eltham had shown they were prepared to advance with the times by erecting that new and commodious hospital, which was built upon the most recent plan, and the architect and the committee who had worked with him were to be congratulated on the result. (Applause.) Everyone in that room would recognise that such a chamber as that they were in would form a very comfortable and healthful resting-place in the days of sickness. (Applause.) He had been anxious to know how they stood financially, for after all finance was the bed-rock of most things. Eltham used to give £100 a year, but this institution would want £400 to £500 a year to keep it up. They had been receiving £100 from collections in churches, £100 from other sources, and £100 in donations which were obtained, he should say, largely through the energy of their president, Mr. Gordon. (Applause.) The list of annual contributions would have to be considerably increased, so that they might receive an additional £100 to £150 per annum. And he would like to say a word to those who would become contributors to the funds of this hospital for the first time. He would remind them of the pleasure and privilege it was to give to an institution of that kind. However much a man possessed, he never knew the true worth of money until he learned to give wisely to works of charity. If there were people in Eltham so selfish as to deny themselves the privilege of giving to this institution, let them keep in that condition if they would. Here they had a cause to give to which was ennobling in itself. It combined self-help with charity, and was at the same time free from any objection which any reasonable person could take. Therefore, those who did not give must take upon themselves the responsibility of not only not having the pleasure that they would thus secure, but, when their own hour of sickness came, they might reflect that they were benefiting by the medical or surgical skill and trained nursing which had been brought to them by the hospitals which they had refused to help to support. The public of Eltham were greatly indebted to Mr. Gordon, and the committee working with him, for undertaking the erection of this hospital, and those who had taken any part in securing its erection had shown great courage and great foresight. (Applause.) Sir Henry concluded: It is now my pleasing duty to declare this hospital open, and to wish that God's blessing may rest upon it, upon those who minister and those who suffer in it, and that every year may endear it to the people of Eltham, and add to its prosperity and usefulness. (Loud cheers.)

Colonel Gordon then asked Sir Henry's acceptance of a silver key, in case, as a small memento of the occasion, at the same time remarking that he was afraid it would not open any of the doors of that building. (Laughter.)

Sir Henry Burdett: I am sure I am very much indebted to you for this gift, which I shall always cherish. If it is a key which will not fit any lock, let us hope it is a key which will fit the lock of our hearts and unite us in affectionate regard for all time. (Applause.)

The Rev. E. J. Penford (Congregational minister), and the Rev. Father Martin seconded a proposition of success to the hospital's work, after which Mr. T. W. Mills (secretary) moved, and Mr. David French seconded, a vote of thanks to the ladies who had been instrumental in doing so much good work in connection with the old hospital and in starting the new one; and the formal programme was completed by the proposition of a vote of thanks to Sir Henry Burdett by Dr. Jeken, seconded by the Rev. G. P. Viner, to which Sir Henry briefly returned thanks. A vote of thanks to the architect was proposed by Dr. Mitchell, seconded by Colonel Gordon, and acknowledged by Mr. A. B. Hutchings. The pleasant proceedings then closed with the hearty singing of the National Anthem, one of the objects of erecting the hospital having been to celebrate the Diamond Jubilee of Her Majesty the Queen.

### THE MENTALLY AFFLICTED AT LEWISHAM.

On December 6th Mr. John Penn, M.P., opened the new building which has just been added to the Lewisham Infirmary for the reception of patients mentally afflicted. Mr. Cuthbert Wilkinson took the chair, and in a short speech gave the reasons for the action of the Guardians in this matter. Primarily, because early treatment is imperative if there is to be any hope of cure in these cases; and secondly, because the stigma of having been in an asylum prevented those who bore it obtaining employment afterwards. It was the duty, therefore, of the Guardians to provide for these patients to the best of their ability, and a system which cured two out of every three patients must of necessity be a very great saving to the ratepayers.

The Chairman complimented the ratepayers upon possessing the finest infirmary in London, and the officials upon the zeal, efficiency, and success with which they discharged their duties, and to whose co-operation the success was due.

Mr. W. Brown, chairman of the Board of Guardians, then gave a description of the improved provisions now made for the insane, describing the methods pursued at Maidstone and at Salisbury asylums, to which they sent their lunatics. He compared private asylums with those supported by the public purse to the disadvantage of the former, because it was profitable to the former to retain their patients as long as possible, and to the latter to cure them. The old lunatic wards at Lewisham are now used as carpenters' shops, and he considered they were bad enough to send the patients mad. He pointed out that it was also cheaper to provide plenty of accommodation for their infirm, as when the number of inmates in the infirmary did not exceed that for which they were certified by the Local Government Board, they received a maintenance allowance of 9d. a day per patient, which was lost when the number of inmates exceeded that limit. They also received a grant from the Metropolitan Asylums Board, thus leaving only the buildings and their preservation to the charge of the local rates.

Dr. Toogood, the medical superintendent, gave it as his opinion that lunatics in the initial stage of their illness were better treated by general practitioners than by specialists. Often physical causes lay at the root of mental disturbance, and with the healing of the body the mind recovered its balance. He mentioned a scheme promoted by the Charity Organisation Society proposing that the London County Council should erect receiving houses for this class of patient. He maintained that it would be no easy matter to provide 2,000 beds, and that the computed cost could not be less than a quarter of a million pounds, whilst, let them call these houses by whatsoever fanciful name they would, in the eyes of the public they would be lunatic asylums, and the stigma attached to anyone confined therein even a few days would materially affect the possibility of their getting permanent employment afterwards. Patients were carefully attended in these wards for fourteen days, and so successful was the immediate care that only one-third had to be drafted on to asylums.

Mr. John Penn, M.P., after disclaiming any knowledge of the subject of the proper treatment of the insane, noted one or two important points in the three previous addresses. The one was the stigma attaching to those unfortunate enough to be sent to asylums, and the value of a provision that reduces this number. He referred to "Valentine Vox" as a book written for the purpose of putting down private asylums, and as having succeeded in its aim, awaking the public conscience, and thus helping forward their present humane treatment, which he had seen exemplified in Government institutions. He congratulated the Guardians on following so closely the most enlightened methods of treating the mentally afflicted, and finally expressed his own gratification upon being invited to be present and upon the welcome accorded him. He formally declared the new building open, and heartily wished that it might prove a blessing.

The new building stands between the workhouse and infirmary, but it belongs entirely to the latter institution and is under the control of the medical superintendent and his staff. It consists of two large oblong wards for eight beds each, with separate entrance, and having its own bath and lavatory accommodation, padded room, kitchen, and isolation room. They are heated by Shorland's stoves placed in the centre of the wards, and strongly guarded. The ventilation is carefully provided for by windows, inlets of fresh air, and flues to carry off the foul air. The walls are "adamant cement," and painted cheerfully in two tints; the floors are pitch pine wooden blocks; the wood work varnished pine. The wards are lofty, roomy, light, airy, and cheerful, whilst the furnishing is simple and tasteful. The padded rooms are octagonal in shape, rubber lined, and well lighted; they are fitted with spring bolts and observation windows. The whole cost has been £3,703, and a loan for this sum will be arranged with the London County Council. A large number of people were present at the ceremony, and numerous letters of regret were read from those unable to attend. The infirmary was open to inspection, and the interest evinced by the residents in the neighbourhood was much appreciated by those who had striven to make the institution thoroughly efficient.

